# Large primary splenic cyst: A laparoscopic technique

**DOI:** 10.4103/0972-9941.51315

**Published:** 2009

**Authors:** M Geraghty, I Z Khan, K C Conlon

**Affiliations:** Department of Surgery, Professorial Surgical Unit, Trinity College Dublin and Adelaide, Meath and National Children's Hospital, Tallaght, Dublin 24

**Keywords:** Fenestration, laparoscopic, splenic cyst

## Abstract

Splenic cysts are rare lesions with around 800 cases reported in the world literature. Traditionally splenectomy was the treatment of choice. However, with the recognition of the important immunological function of the spleen, new techniques to preserve splenic function have been developed. This case emphasizes that in selected cases splenic preservation is appropriate.

## INTRODUCTION

Cystic lesions of the spleen include benign cysts, neoplasms and abscesses. Splenic cysts have been classified by Martin *et al*.[1] Type I cysts are true (primary) cysts with a cellular lining of parasitic or non parasitic origin. Non parasitic type I splenic cysts are further classified as congenital or neoplastic. Type II cysts are false (secondary) cysts without a cellular lining, the most commonly found following blunt trauma to the spleen.

Non parasitic splenic cysts are most common in Europe and North America; splenic cysts of parasitic origin are more common in Africa and Central America.[2] The prevalence of splenic cysts has increased recently secondary to increased detection with computerized tomography and the non-operative management of certain types of splenic injury.[3] The true incidence of splenic cysts is unknown and as yet there is no evidence-based management. With a move away from splenectomy due to the spleen's important immunological function against encapsulated bacteria, splenic preserving surgery (open and minimally invasive) is preferred.

## CASE REPORT

A 38-year-old female underwent investigation for upper abdominal pain and abnormal bowel habit. Physical and haematological examinations were unremarkable. She had an oesophagogastroscopy (OGD) and colonoscopy. At gastroscopy an impression of a sub-epithelial lesion bulging inwards towards the fundus of the stomach was found. Endoscopic ultrasound was performed which demonstrated a large cyst in the upper abdomen. Computerized tomography with oral and intravenous contrast confirmed a 10 cm × 7 cm cystic mass arising from the upper pole of the spleen. There was no septation and its contents appeared homogeneous. In view of the potential complications and an active life style it was decided to proceed to splenic fenestration.

**Figure 1:**
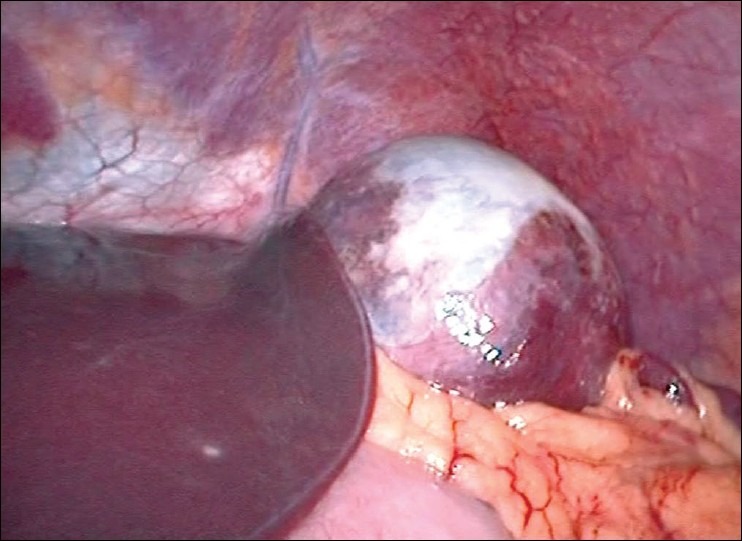
Splenic cyst

**Figure 2:**
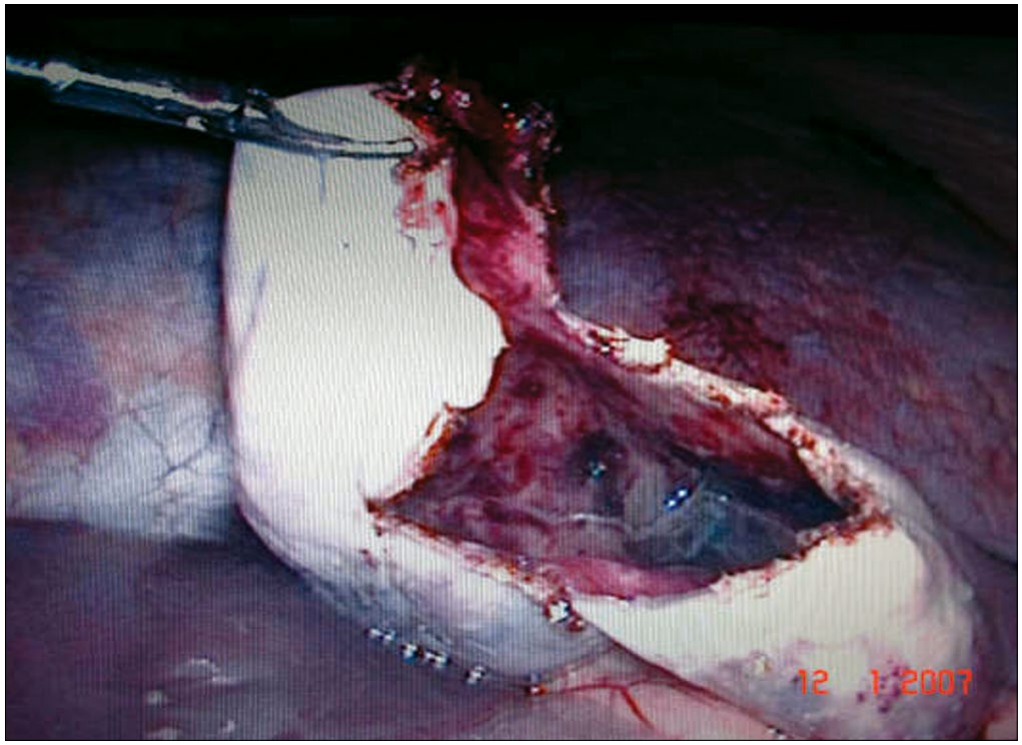
Fenestrated cyst wall

**Figure 3:**
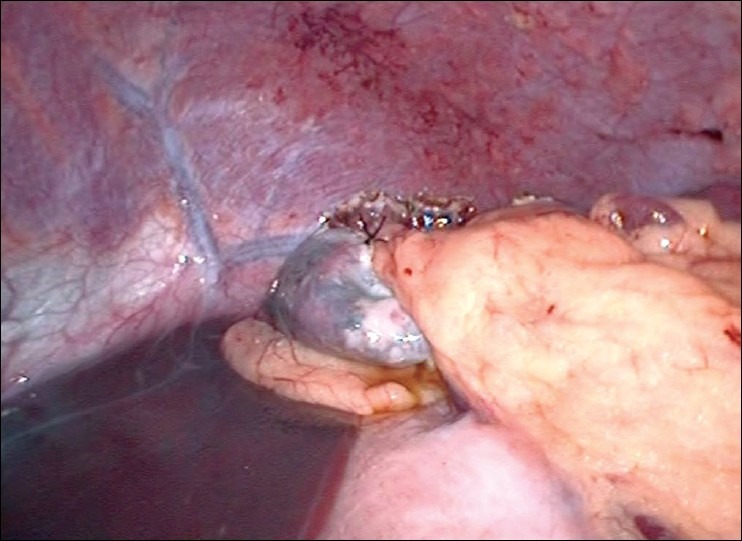
Omental packing in fenestrated cyst

After standard preoperative maneuvers, entry to abdomen was made via periubumbilical incision and a modified Hassan's open technique. A 30-degree laparoscope was inserted and the splenic cyst was identified [Figure 1]. Under direct vision a 10 mm and two 5 mm ports were inserted in the epigastrium, left and right upper quadrants, respectively. The simple nature of the cyst was confirmed by the aspiration of straw-colored fluid. Using an ultrasonic dissector (Sonosurg™ Olympus, Tokyo, Japan), the cyst wall was opened and resected to the splenic parenchyma [Figure 2]. The area of resection was inspected for haemostasis. An omental flap was constructed and placed into the cyst cavity [Figure 3]. The patient had an uneventful postoperative recovery and was discharged within twenty four hours. Histopathology showed a fibrous cyst wall consistent with a simple cyst, with no evidence of parasitic origin. At follow-up, the patient remains asymptomatic.

## DISCUSSION

The true incidence of splenic cysts is unknown and management is controversial. Blunt trauma to the upper abdomen is the commonest cause of secondary cyst formation. It is considered responsible for 75% of secondary splenic cysts.[4] Splenic cysts may remain asymptomatic in 30 to 60% of patients.[5] Splenic cyst formation following blunt abdominal trauma is thought to occur following haematoma formation with resultant resorption and serous fluid collection.[3] The most common symptoms include left upper quadrant pain, nausea and vomiting secondary to compression of the stomach. They may also be discovered following the complications of rupture, intracapsular haemorrhage or infection. Indications for operative intervention include cysts with a diameter >5 cm and those which are symptomatic. Cysts with a diameter >5 cm are more likely to rupture resulting in life threatening haemoperitoneum.

As the indication for an operation is often relative, the choice of surgical technique is of special interest. Operative intervention aims to eradicate the cyst and prevent recurrence. Operative methods include both open and laparscopic techniques. In the past, splenectomy was thought to be the only management.[6,7] However, splenic preserving surgery has the benefit of avoiding the lifelong risk of the potentially fatal overwhelming post splenectomy sepsis (OPSI). Post splenectomy patients have a lifetime risk of 5% for developing OPSI, which carries a mortality of 38-69%.[8] Options for splenic preservation include partial splenectomy, cystectomy, and cyst decapsulation. Other methods include drainage techniques using radiological guidance, the incidence of recurrence with this method has been reported to be as high as 100%, and for this reason surgery is considered the treatment of choice.[3]

Salky *et al*, first reported laparoscopic unroofing of a splenic cyst in 1985, this involved opening a 3 cm window in a post traumatic splenic cyst, and they reported a good outcome with no radiological evidence of cyst recurrence after 8 months.[9] Robertson *et al*, reviewed 32 cases following laparoscopic intervention for splenic cysts and found the recurrence rate to be 22%, of those that recurred only 3% required further operative intervention for cyst recurrence.

To minimize the risk of recurrence it is felt that the majority of cyst wall should be removed; this is normally to the splenic parenchyma as was performed in our case. An omental flap was created to maintain drainage and prevent recurrence. The use of the ultrasonic dissection reduces the incidence of haemorrhage from the walls thus significantly minimizing one of the known complications of this technique.

Choice of procedure also depends on the location of the cyst; those located on the anterior surface are suitable for laparocopic fenestration. Cysts in the posterior surface of the spleen are found to be more difficult for a laparoscopic treatment since the spleen has to be widely mobilized. In the case of a centrally located splenic cyst, a laparoscopic procedure should not be attempted and an open partial splenic resection is recommended.[10]

The true incidence of splenic cysts is unknown, splenic preservation has become the accepted treatment for blunt trauma to the spleen. Laparoscopy provides the advantage of decreased pain, quicker recovery, and shorter hospital stay. The technique of laparoscopic fenestration of splenic cysts described in this paper is a safe method for elimination of splenic cysts and preservation of splenic function.
